# Factors associated with COVID-19 booster vaccine willingness among migrants from the Eastern Mediterranean living in Australia: a cross-sectional study

**DOI:** 10.1186/s12889-022-14608-5

**Published:** 2022-11-28

**Authors:** Keeley Allen, Stephen B. Lambert, Aidan Yuen, Davoud Pourmarzi

**Affiliations:** 1 National Centre for Population Health and Epidemiology, Australian National University, Canberra, ACT Australia; 2 National Centre for Immunisation Research and Surveillance, Westmead, NSW Australia

**Keywords:** Migrant health, COVID-19, Vaccine, Booster, Hesitancy, Health behaviour

## Abstract

**Background:**

Migrants have been disproportionally affected by COVID-19 in Australia. Vaccination against COVID-19 is a key pillar of Australia's public health response, but little is known about the willingness to receive booster vaccinations among migrants. This study aimed to assess the factors associated with a willingness to receive a COVID-19 booster vaccine among migrants living in Australia born in the World Health Organization’s Eastern Mediterranean Region (EMRO).

**Methods:**

A cross-sectional survey was conducted from September to November 2021 (*n* = 300). Participants were questioned on booster vaccine willingness, sociodemographic characteristics, COVID-19 vaccine information needs and sources, and perceptions of COVID-19 vaccines. Univariate and multivariate logistic regression were used to assess factors associated with booster willingness.

**Results:**

Most respondents (87%) had received two doses of COVID-19 vaccine, of which 81% were willing to receive a booster dose. About half of the participants reported high or very high needs for receiving information about “COVID-19 vaccines’ safety monitoring in Australia”, “COVID-19 vaccines protection against illness”, “Safety of COVID-19 vaccines used in Australia”, and “The Australian COVID-19 vaccination program”. People who were willing to receive a boost dose had significantly higher self-estimated knowledge of COVID-19 vaccines, confidence in COVID-19 vaccines and trust in the Australian government’s vaccine recommendations, and perceived COVID-19 as a greater risk compared to those who were unsure/hesitant. Both groups reported similar perceptions of their personal risks from COVID-19 but diverged on their views of COVID-19 as a broader health problem. There were no statistically significant differences between the two groups in terms of channels used to find information about COVID-19 vaccines. Factors associated with willingness to receive a COVID-19 booster vaccine in the multivariate logistic regression were age (aOR 1.07 95% CI 1.02–1.12), and no exposure to concerning news about COVID-19 vaccines (aOR 3.71 95% CI 1.51–9.09).

**Conclusion:**

Vaccine acceptance and reported booster willingness was high. The results suggest the news and information seen may impact willingness to receive booster doses, even among those who have already received doses of COVID-19 vaccine. Addressing vaccine concerns and transparent communication about uncertainty should be a priority in the current and in future pandemics.

## Introduction

Vaccinations are fundamental to controlling the spread of COVID-19 and protecting populations. Australia has seen significant uptake of primary vaccine doses with over 76% of the population having received at least two doses of COVID-19 vaccines [1]. High vaccine uptake has stemmed from widespread community acceptance, targeted vaccine mandates to engage in aspects of public life until a threshold of adult vaccinations was met, and vaccination mandate for specific industries’ workforce [2]. Waning immunity from vaccines and the emergence of new variants have seen booster doses become a pillar of public health responses worldwide. In Australia, people who were severely immunocompromised were recommended to receive a third dose (considered part of the primary course) of a COVID-19 vaccine in early October 2021 [3]. A third (booster) dose of COVID-19 vaccine was recommended from 8 November 2021 for the general population aged 18 years and older who received their primary doses at least six months prior [4]. However, a recent Australian survey showed that the availability of booster vaccines and receiving primary doses of COVID-19 vaccines are not indicative of a willingness to receive a COVID-19 booster vaccine for all populations [5].

Worldwide, migrants have been disproportionately affected by the COVID-19 pandemic, amplifying existing health and social disparities [6–9]. Several factors make migrants more vulnerable to COVID-19 and less engaged in public health measures, compared with locally born populations. Migrants have had a higher risk of exposure to the pathogen as this population generally fills roles in essential services more likely to require face-to-face contact with the public [7–9]. Migrants generally face additional accessibility barriers to health care that have been exacerbated by the COVID-19 pandemic, such as unmet language requirements, limited access to culturally appropriate services, and limited entitlement to subsidised health care [7, 10–13]. In addition, migrant communities have had limited input and engagement in the development of public health messages to promote COVID-19 vaccinations, contributing to lower vaccination coverage against COVID-19 [10]. As a result, migrants have higher hospitalisation and death rates than locally born populations [7, 14, 15]. Recent analysis of COVID-19 death data in Australia revealed that migrants had an age-standardised death rate over 2.5 times higher than locally born populations, and migrants born in Middle Eastern countries have been subject to the highest age-standardised death rate at 29.3 deaths per 100,000 population per year in Australia [16]. An estimated 7.6 million Australian residents were born overseas, accounting for approximately 30% of the total population [17]. Migrants have the right to health and for them to successfully contribute to the society, it is fundamental that their health is protected and that they are included in health system responses [18].

Understanding migrants’ willingness to receive a booster dose and the factors affecting their decision can help inform interventions to prevent COVID-19 and promote health equity. Knowledge about the acceptance of COVID-19 booster vaccines among migrant populations in Australia is currently lacking [19–21]. With this study we aimed to understand willingness to receive a COVID-19 booster and associated factors among migrants from the World Health Organization’s Eastern Mediterranean (EMRO) region living in Australia.

## Methods

### Research design and sampling

We conducted a cross-sectional study using an anonymous online questionnaire available to participants between 27 September 2021 and 30 November 2021. During this period, the primary doses of COVID-19 vaccines were becoming available to the adult population of Australia. Various restrictions and vaccine mandates were in place, with Victoria, New South Wales, and the Australian Capital Territory subject to stay-at-home orders for some part of the study period [2]. Although booster doses became available during the study period on 8 November 2021, discussion among health authorities and the public about the need for a booster dose had started months before data collection for this project, as some countries had started offering booster does to their population [3, 4].

Individuals were eligible to participate if they were aged 18 years and older, currently residing in Australia, and born in one of the following countries which make up the EMRO region: Afghanistan, Bahrain, Djibouti, Egypt, Iran, Iraq, Jordan, Kuwait, Lebanon, Libya, Morocco, Oman, Pakistan, Palestine, Qatar, Saudi Arabia, Somalia, Sudan, Syria, Tunisia, United Arab Emirates, or Yemen. Participants born in Algeria were not included in this study as the country falls within the World Health Organization's African Region.

Recruitment of participants relied on convenience (non-random) sampling. The researchers disseminated the study flyer through social media platforms (primarily Facebook, Twitter, and Instagram), approaching community leaders and social media community groups supporting migrants from the targeted countries in Australia. The flyer was also distributed electronically through non-government organisations that provide support for migrant populations in Australia.

### Study questionnaire and data collection

The questionnaire was developed based on a review of literature and using the Risk Information Seeking and Processing model [22], and the three Cs model of vaccine hesitancy: confidence, complacency, and convenience [23]. The questionnaire was designed and administered using Qualtrics software (Qualtrics, Provo, UT, USA, version November 2021) hosted by the Australian National University. The questionnaire was developed in English and translated into Arabic and Persian (Farsi), and participants could select which language to view. Prior to release, the online questionnaire was reviewed by five individuals from the targeted communities to ensure its clarity and simplicity.

The questionnaire consisted of six domains. The first domain collected sociodemographic data, including age, gender, jurisdiction of residence, country of birth, years lived in Australia, highest level of educational attainment, and health profession status. Health profession status was defined as employment or studying a clinical (nursing, medical, or allied health) or public health profession. Participants were asked to describe their occupation or area of study. Participants were also asked about their current COVID-19 vaccination status.

The second domain asked participants to subjectively assess their level of knowledge of COVID-19 vaccines and the Australian COVID-19 vaccination program on a 0–100 continuous scale. The third domain asked participants to indicate their level of need for information across five different aspects of COVID-19 vaccines, and the Australian COVID-19 vaccination program on a 5-point scale (very low need, low need, moderate need, high need, very high need).

The fourth domain measured a participant’s confidence in COVID-19 vaccines (two items), trust in the Australian government’s decision making around COVID-19 vaccines (one item) and risk perception of COVID-19 (four items). Each question was asked on a 5-point Likert scale (strongly disagree, disagree, neither agree nor disagree, agree, and strongly agree).

The fifth domain focused on information channels participants used to collect information on COVID-19 vaccines. Participants were able to select multiple options, including official sources (such as government and health care professionals) in Australia, official sources in their home country, and unofficial sources such as mass media or friends and family.

The final domain measured a participant’s willingness to receive a COVID-19 booster vaccine. This domain formed the outcome variable for this study and was only available to participants who indicated they either had already received two COVID-19 vaccine doses or had received one dose and planned to receive a second dose. Participants were asked on a 5-point Likert scale “If it is recommended, how likely are you to get a COVID-19 vaccine again next year?”. Due to the small number of participants selecting “unlikely” and “extremely unlikely”, responses, were combined into a binary variable, with participants responding, “extremely likely” or “likely” considered willing, and participants responding, “neither likely nor unlikely”, “unlikely”, or “extremely unlikely” considered unsure/hesitant.

### Data analysis

Descriptive analyses were conducted to describe study participants. Frequencies and proportions were calculated for categorical variables, and continuous variables were summarised by mean and standard deviation. Median values and ranges were also calculated for age and years lived in Australia; in anticipation that the data would follow a non-normal distribution. Ten-year age groups were created for descriptive analyses and age was entered as a continuous variable for univariate and multivariate analyses. Those who identified as non-binary or preferred not to disclose their gender were excluded from cross-tabulations, univariate, and multivariate analyses due to low numbers. Jurisdiction of residence was grouped based on stay-at-home order experiences. New South Wales and the Australian Capital Territory residents were combined as respondents who experienced extended stay-at-home orders in 2021, Victorian residents experienced extended stay-at-home orders in 2020 and 2021, and all other jurisdictions were combined as regions of Australia experiencing relatively minimal periods of stay-at-home orders. Cross-tabulation analyses were prepared to assess the association of categorical variables with willingness to receive a booster vaccine using the chi-squared test. Continuous variables were analysed using the Wilcoxon rank sum test. Univariate analyses were conducted to calculate unadjusted odds ratios (ORs). Variables that returned a *p*-value ≤ 0.2 were included in the multivariate binary logistic regression as risk factors/exposures and potential confounders [24]. Age and gender were included as a priori confounders in the multivariate binary logistic regression regardless of the statistical significance found in the univariate analysis. Statistical significance for adjusted ORs in the multivariate binary logistic regression was set at a *p*-value < 0.05 and where the 95% confidence interval (CI) did not contain 1. All data were analysed in Stata (version 17.0).

### Ethical considerations

The protocol for this study was approved by the Australian National University Human Research Ethics Committee (2021/224). Participation was voluntary and informed consent was obtained at the start of the questionnaire. Only questions that related to the inclusion criteria (i.e., age and country of birth) and health professional status were compulsory. Participants had the option to enter a prize draw to win one of ten $100 grocery vouchers. Contact details of participants who elected to enter the draw were collected and kept in a separate online form from questionnaire responses.

## Results

The questionnaire was attempted by 302 participants. One respondent was younger than 18 years of age and one respondent did not complete all questions regarding inclusion criteria. Inclusion criteria were met by 300 respondents and 216 responded to the outcome variable (i.e., willingness to receive a COVID-19 booster vaccine). Approximately half of the participants were male (52.0%), the median age of respondents was 39 years (range: 18–74 years, IQR: 34–48 years) and the median time lived in Australia was seven years (range: 0.2–40 years, IQR: 4.3–11 years) (Table 1). Most respondents (71%) were university-educated and were not studying or working in a health profession (87%) (Table 1). Responses were received from migrants born in all EMRO countries except for Djibouti and Qatar.

**Table 1:** Sociodemographic characteristics of study participants

Characteristics		Number	Percentage
Gender	Man or male	156	52.0%
	Woman or female	138	46.0%
	Non-binary	1	0.3%
	Prefer not to say	5	1.7%
Age group (years)	18–24 years	17	5.7%
	25–34 years	67	22.3%
	35–44 years	122	40.7%
	45–54 years	47	15.7%
	55–64 years	30	10.0%
	65 + years	17	5.7%
Jurisdiction of residence	Australian Capital Territory	55	18.3%
	New South Wales	86	28.7%
	Northern Territory	1	0.3%
	Queensland	51	17.0%
	South Australia	9	3.0%
	Tasmania	5	1.7%
	Victoria	73	24.3%
	Western Australia	20	6.7%
Years lived in Australia	0–4 years	98	32.7%
	5–9 years	111	37.0%
	10 + years	91	30.3%
Educational attainment	Have not completed high school	27	9.0%
	Completed high school	34	11.3%
	Vocational certificate	27	9.0%
	Associate degree	33	11.0%
	Bachelor degree	84	28.0%
	Master or Doctoral degree	95	31.7%
Health profession status	Yes	38	12.7%
	No	262	87.3%
Vaccination status at time of questionnaire	Have not received a COVID-19 vaccine	10	3.9%
	Had received one vaccine, and not intending to have a second	4	1.6%
	Had received one vaccine, and intending to have a second	19	7.4%
	Had received two doses	224	87.2%
Country of birth	Iran	84	28.0%
	Iraq	37	12.3%
	Pakistan	30	10.0%
	Syria	30	10.0%
	Afghanistan	24	8.0%
	Other EMRO country	95	31.7%

Most respondents (87%) reported receiving two doses of a COVID-19 vaccine at the time they completed the questionnaire. Of the 10 respondents who had not received a COVID-19 vaccine, four were extremely likely or somewhat likely to receive a COVID-19 vaccine, four were neither likely nor unlikely, and two were somewhat unlikely or extremely unlikely to receive a COVID-19 vaccine.

On the scale from 0 to 100, the mean self-estimated knowledge of COVID-19 vaccines was 71.3 out of 100 (± 24.6) and the mean self-estimated knowledge of the Australian COVID-19 vaccination program was 70.3 ± 27.1. About half of the participants reported high or very high needs for receiving information about “COVID-19 vaccines safety monitoring in Australia”, “COVID-19 vaccines protection against illness”, “Safety of COVID-19 vaccines used in Australia”, and “The Australian COVID-19 vaccination program” (Table 2).

**Table 2:** Level of information need of study participants regarding COVID-19 vaccines and the Australian vaccination program

Area of information need	Very low n (%)	Low n (%)	Moderate n (%)	High n (%)	Very high n (%)
Safety of COVID-19 vaccines used in Australia	10.8%	11.5%	28.2%	27.9%	21.6%
COVID-19 vaccines protection against illness	7.7%	12.9%	29.0%	32.5%	17.8%
Ingredients of the COVID-19 vaccines used in Australia	13.0%	18.0%	29.2%	27.8%	12.0%
The way that COVID-19 vaccines work	10.3%	14.6%	30.3%	31.7%	13.2%
COVID-19 vaccines’ safety monitoring in Australia	7.5%	17.1%	23.9%	32.9%	18.6%
The Australian COVID-19 vaccination program	8.6%	11.9%	30.6%	32.4%	16.6%

Among participants who either had received two COVID-19 vaccine doses or had received one dose and planned to receive a second dose, 176/216 (81.4%) indicated they were willing to receive a COVID-19 booster vaccine. Two thirds (66.1%, 143/216) of respondents indicated they were “extremely likely” to receive a COVID-19 booster vaccine. Among those who were unsure/hesitant, most respondents were “neither likely nor unlikely” to receive a booster dose (87.5% of unsure/hesitant respondents, 16.2% of all respondents).

Table 3 shows aspects of information seeking behaviours by willingness to receive a COVID-19 booster dose. Respondents who were willing to receive a COVID-19 booster vaccine had a higher mean self-estimated knowledge of COVID-19 vaccines (75.04 ± 22.54) compared to respondents who were unsure/hesitant (60.50 ± 28.70) (*P* < 0.01). Overall, respondents who were willing to receive a COVID-19 booster had greater scores in confidence in COVID-19 vaccines and trust in the Australian government’s vaccine recommendations domain, and perceived COVID-19 as a greater risk, compared with those who were unsure/hesitant about receiving a booster dose. Both groups reported similar perceptions of their personal risks from COVID-19 but diverged in their views of COVID-19 as a broader health problem. There were no statistically significant differences between the two groups in terms of channels used to find information about COVID-19 vaccines. A greater proportion of respondents who were willing to receive a COVID-19 booster vaccine reported they had no recent exposure to concerning COVID-19 vaccine news and did have exposure to news about COVID-19 vaccines that made them feel confident.

**Table 3:** Information seeking behaviours and willingness to receive a COVID-19 booster vaccine among study participants

Domains		COVID-19 booster willingness		Total (SD)	Test statistic	*p*-value
		Willing Mean ± SD / N (%)	Unsure/hesitant Mean ± SD / N (%)			
Estimated knowledge	Estimated knowledge of COVID-19 vaccines (0–100)	75.04 ± 22.54	60.50 ± 28.70	72.35 ± 24.39	-2.901^a^	< 0.01*
Confidence score	The COVID-19 vaccines used in Australia are effective in protecting us	4.35 ± 0.73	3.65 ± 1.00	4.22 (0.83)	-4.308^a^	< 0.01*
	The COVID-19 vaccines used in Australia are safe	4.22 ± 0.82	3.60 ± 1.01	4.10 ± 0.89	-3.757^a^	< 0.01*
	Total	4.28 ± 0.70	3.63 ± 0.90	4.16 ± 0.78	-4.390^a^	< 0.01*
Risk perception score	COVID-19 is a serious health problem around the world	4.56 ± 0.75	4.03 ± 1.00	4.46 ± 0.83	-3.516^a^	< 0.01*
	COVID-19 is a serious health problem in Australia	4.36 ± 0.83	4.08 ± 0.87	4.31 ± 0.84	-2.090^a^	0.04*
	I'm at risk of getting COVID-19	3.59 ± 1.09	3.49 ± 1.25	3.57 ± 1.12	-0.395^a^	0.69
	If I get COVID-19 I could be seriously ill	3.81 ± 1.01	3.74 ± 0.94	3.80 ± 1.00	-0.534^a^	0.59
	Total	4.08 ± 0.66	3.84 ± 0.60	4.03 ± 0.83	-2.394^a^	0.02*
Trust	I trust the Australian government recommendations for COVID-19 vaccines	4.29 ± 0.83	3.73 ± 1.04	4.18 ± 0.89	-3.560^a^	< 0.01*
Used official Australian information channel	Yes	147 (83.1%)	34 (82.9%)	181 (83.0%)	< 0.001^b^	0.99
	No	30 (16.9%)	7 (17.1%)	37 (17.0%)		
Used official home country information channel	Yes	33 (18.6%)	11 (26.8%)	44 (20.2%)	1.384^b^	0.24
	No	144 (81.4%)	30 (73.1%)	174 (79.8%)		
Used unofficial information channel	Yes	145 (81.9%)	31 (75.6%)	176 (80.7%)	0.852^b^	0.36
	No	32 (18.1%)	10 (24.4%)	42 (19.3%)		
In recent months, I have heard some news about COVID-19 vaccines that made me confident	True	137 (78.7%)	21 (51.2%)	158 (73.5%)	12.895^b^	< 0.01*
	False	37 (21.3%)	20 (49.8%)	57 (27.5%)		
In recent months, I have heard some news about COVID-19 vaccines that made me concerned	True	65 (36.9%)	28 (68.3%)	93 (43.9%)	13.355^b^	< 0.01*
	False	111 (63.1%)	13 (31.7%)	124 (57.1%)		

^a^Wilcoxon rank sum test statistic

^b^Chi-squared test statistic

Statistically significant results are shown with an asterisk (*)

The results of the univariate and multivariate analyses are shown in Table 4. Exposure to confidence-inducing news about COVID-19 vaccines (OR 3.68 95% CI: 1.79–7.54), no exposure to concerning news about COVID-19 vaccines (OR 3.51 95% CI: 1.70–7.29), higher educational attainment (OR 2.34 95% CI: 1.13–4.84), greater confidence in COVID-19 vaccines (OR 2.76 95% CI: 1.75–4.34), perceiving COVID-19 as a risk (OR 1.74 95% CI:1.02–2.97), trust in the Australian government’s approach to vaccines (OR 1.85 95% CI: 1.30–2.65), increasing age in years (OR 1.07 95% CI: 1.03–1.11), and higher mean self-estimated knowledge of COVID-19 vaccines (OR 1.02 95% CI 1.01–1.04) were univariately associated with willingness to receive a COVID-19 booster vaccine. The strongest associations were evident regarding the news or subject of information a respondent was exposed to regarding COVID-19 vaccines. No significant differences between the groups were seen by gender, health profession status, years lived in Australia, or information channel used.

**Table 4:** Univariate and multivariate binary logistic regression analysis of factors associated with willingness to receive a COVID-19 booster

Characteristic		Unadjusted odds ratio (OR)	95% CI	*p*-value	Adjusted odds ratio (aOR)	95% CI	*p*-value
Age	Age (years)	1.07	1.03–1.11	< 0.01	1.07	1.02 –1.12	0.01*
Gender	Man or male	REF	-	-	REF	-	-
	Woman or female	1.15	0.58–2.31	0.69	1.35	0.58–3.12	0.47
Jurisdiction of residence	NSW/ACT	REF	-	-	REF		
	Victoria	0.41	0.17–0.95	0.04	0.52	0.19–1.44	0.21
	Other	0.59	0.25–1.36	0.22	0.60	0.22–1.81	0.41
Educational attainment	Non-university	REF	-	-	REF	-	-
	University	2.34	1.13–4.84	0.02	2.35	0.96–5.75	0.06
Health profession status	No	REF	-	-	-	-	-
	Yes	6.76	0.89–51.38	0.07	-	-	-
Years lived in Australia	0–9 years	REF	-	-	REF		
	10 years +	1.77	0.77–4.09	0.18	0.82	0.29–2.33	0.71
Estimated knowledge	Estimated knowledge of COVID-19 vaccines	1.02	1.01–1.04	< 0.01	1.01	0.99–1.03	0.23
Confidence	Confidence score	2.76	1.75–4.34	< 0.01	1.92	0.95–3.87	0.07
Risk perception	Risk perception score	1.74	1.02–2.97	0.04	1.50	0.77–2.90	0.23
Trust	Trust score	1.85	1.30–2.65	< 0.01	1.00	0.58–1.70	0.99
Used official Australian information channel	No	REF	-	-	-	-	-
	Yes	1.03	0.42–2.55	0.95	-	-	-
Used official home country information channel	No	REF	-	-	-	-	-
	Yes	0.61	0.28–1.34	0.22	-	-	-
Used unofficial information channel	No	REF	-	-	-	-	-
	Yes	1.50	0.67–3.38	0.33	-	-	-
In recent months, I have heard some news about COVID-19 vaccines that made me confident	No	REF	-	-	REF	-	-
	Yes	3.68	1.79–7.54	< 0.01	1.50	0.57–3.98	0.82
In recent months, I have heard some news about COVID-19 vaccines that made me concerned	Yes	REF	-	-	REF	-	-
	No	3.51	1.70–7.29	< 0.01	3.71	1.51–9.09	< 0.01*

Statistically significant results are shown with an asterisk (*)

A total of 209 respondents were included in the multivariate binary logistic regression analysis. After multivariate binary logistic regression analysis, only no exposure to concerning news about COVID-19 vaccines (aOR 3.71 95% CI 1.51–9.09) and age (aOR 1.07 95% CI 1.02–1.12) were significantly associated with willingness to receive a COVID-19 booster vaccine.

## Discussion

Overall, this study found high COVID-19 vaccine uptake and a high willingness to receive a booster vaccine amongst EMRO migrants in Australia. Willingness was greatest among older respondents and those respondents who had not been exposed to concerning news about COVID-19 vaccines recently. While participants reported moderate knowledge of COVID-19 vaccines and the Australian vaccination program, the highest levels of information needs were related to the safety and effectiveness of the COVID-19 vaccine.

Safety was a key domain of information need among study participants. Concerns regarding the safety of new vaccines and the structures in place to monitor these new vaccines have been found to be significant factors affecting COVID-19 booster vaccine acceptance elsewhere [10, 25–27].

Willingness to receive a COVID-19 booster vaccine among the study population was high. These findings are similar to studies assessing willingness for booster vaccines in Europe and North America [25–30]. This is a reassuring finding for public health authorities, as the success of a vaccination program relies in part on the willingness of a population to accept available vaccines. The high primary dose uptake and willingness to receive a booster dose among our study population may be attributed to community engagement programs, which improved accessibility for different communities by providing information in several languages in written and oral formats and outreach vaccination clinics for specific communities [31, 32].

The high vaccine uptake and willingness to receive a booster vaccine, alongside the elevated need for information on vaccine safety and effectiveness, may be in part due to the presence of vaccine mandates across Australian states and territories at the time of the study [2]. The practical considerations of interacting in public life may have outweighed safety concerns for some participants [33, 34]. Further, the emergence of new variants, increase in breakthrough infections, and waning immunity may have affected willingness to receive a booster vaccine and potentially generated concerns about effectiveness in the time since this study was conducted.

This study found older age was positively associated with willingness to receive a COVID-19 booster vaccine. Older individuals may have higher rates of vaccine willingness due to their increased risk of severe illness, hospitalised and mortality from COVID-19. However, the perceived risk of COVID-19 was not associated with the willingness of receiving a booster dose in this study. Age is a significant factor across other studies, with younger populations more likely to be hesitant or unsure [25, 27, 35–37]. If a further booster dose is recommended for all people aged 18 and older as a public health measure to control COVID-19, public health authorities should consider providing targeted messages to younger populations to increase willingness and uptake of COVID-19 booster vaccines.

It is evident from this study that exposure to negative or concerning news can have a significant impact on willingness to receive a COVID-19 vaccine booster. This impact persists regardless of the level of educational attainment, trust in government, and type of information channel used. Previous studies reported that information need, subjective norms, perceived risk, and information channels can affect COVID-19 vaccine willingness, but they did not ask participants explicitly about exposure to the concerning news [12, 25, 26, 28, 29, 33, 34, 37–40]. The effect of exposure to concerning news on willingness to receive a booster dose is an area that needs further research, especially with the introduction of new vaccines. Our finding may be related to explicitly asking respondents about the news they had consumed in recent months. Exposure to news appears to affect willingness to receive COVID-19 vaccines and highlights the importance of developing and promoting meaningful, trustworthy messages to build acceptance of COVID-19 vaccines and combat misinformation.

Uncertainty about new vaccines, the COVID-19 pandemic, and the perception of changing information and advice, can affect how individuals interpret and trust information and public health expertise over time [33, 34, 41, 42]. Ambiguity around an issue such as COVID-19 vaccines is an important issue for the general public and can facilitate the spread of misinformation and rumour [43]. In the time of uncertainty about effectiveness and safety of a vaccine it is important that the health authorities are equipped with the knowledge and skills of uncertainty communication. Designing communications in a way that help people to understand what is known, what is not known, and what actions are underway can be helpful to build trust and help people to make an informed decision. Reactions to health recommendations are dependent on the trustworthiness of the source which is defined by the expertise, honesty, and good intentions of the communicator [42, 44]. Identifying, acknowledging, and understanding people's concerns about vaccines, explaining why vaccination is recommended rather than solely focusing on what an individual should do, and acknowledging information may change as new research is conducted may improve booster uptake.

### Strengths and limitations

This work makes an important contribution to understanding the information needs of migrants residing in Australia and their intentions to receive a COVID-19 booster dose. The Risk Information Seeking and Processing model and the three Cs model of vaccine hesitancy were used in developing the questionnaire which helped develop a comprehensive understanding of factors affecting willingness to receive COVID-19 vaccine booster dose and information seeking behaviours. The content of the questionnaire was specific to the study and was not validated through the Delphi Method or factor analysis. The questionnaire was available in two of the most common languages spoken in the EMRO region and in English, and participants had the option to select the language they preferred. This removed some potential language barriers to participation. However, the questionnaire was not available in all languages spoken in the region, such as Urdu, Pashto and Somali. The Eastern Mediterranean Region does not include all countries, regions and areas of the Middle East and North Africa, potentially excluding individuals with similar lived experiences to the participants of this study. This study relied on non-random sampling, the questionnaire was only available online and largely relied on social media dissemination, potentially limiting participation from members of the target population who do not regularly use the internet or social media. Combined, these factors and the low sample size may introduce selection bias and limit the generalisability of the findings to populations outside this study, including all migrants from the EMRO region living in Australia. Social desirability may have affected responses to some of the questions, including the question about willingness to receive a booster dose. This was minimised by anonymising the questionnaire. Finally, the cross-sectional nature of this study limits the causal relationship interpretation of findings. At the time of data collection, restrictions were in place to control the spread of the Delta variant which may have affected an individual’s willingness to receive booster vaccines.

## Conclusion

Migrants in Australia are from a myriad of cultural, religious, ethnic, and linguistic backgrounds and represent a wide range of experiences in the COVID-19 pandemic. This diversity needs to be considered and incorporated into vaccination education programs. Interventions and information targeting younger adults and improving information sufficiency in COVID-19 vaccines should be used to encourage booster vaccine uptake. Further qualitative research can help to explore the nuances behind the effects of concerning news and available information on willingness to receive a COVID-19 booster vaccine and other new vaccines. It is crucial that public health practitioners are open about what is known and unknown and communicate frequently to alleviate uncertainty. Addressing vaccine concerns should be a priority in the current and future pandemics.

## Data Availability

Data is available from the corresponding author under reasonable request.
